# Pseudoaneurysm of Left proximal Common Carotid Artery following penetrating trauma

**DOI:** 10.12669/pjms.335.13090

**Published:** 2017

**Authors:** Rehana Shaikh, Saba Sohail, Parvez Ahmed Shaikh, Qamar-un-Nisa Nisa

**Affiliations:** 1 Dr. Rehana Shaikh, MBBS, FCPS, FRCR2A. Consultant Radiologist, CT & MRI Centre, Radiology Department, Dow Medical College/Civil Hospital, DUHS, Karachi, Pakistan; 2 Dr. Saba Sohail, MBBS, MCPS, FCPS, PhD. Professor of Radiology, Incharge CT & MRI Centre, Radiology Department, Dow Medical College/Civil Hospital, DUHS, Karachi, Pakistan; 3 Dr. Parvez Ahmed Shaikh, MBBS Resident Radiology, Radiology Department, Dow Medical College/Civil Hospital, DUHS, Karachi, Pakistan; 4 Dr. Qamar-un-Nisa, MBBS, Resident Radiology, Radiology Department, Dow Medical College/Civil Hospital, DUHS, Karachi, Pakistan

**Keywords:** CTA, Delayed presentation, Foreign body, Penetrating trauma, Pseudoanuerysm

## Abstract

A 33-year male with history of penetrating trauma to left upper chest in 2006, presented through Medical unit to Radiology Department with complain of hemoptysis. Chest X-ray showed a soft tissue lesion in left upper lobe with a linear metallic foreign body. Contrast enhanced CT scan of chest and later CTA was performed which showed a saccular aneurysm arising from mediastinal part of left common aortic artery surrounded by thrombosis with a cylindrical linear metallic foreign body. He was planned for endovascular repair with stenting which he could not afford due to financial constraints. He is currently on conservative follow up. Vascular lesions can be serious complications resulting from blunt or penetrating trauma, when presenting with hemoptysis due to pseudaoneurysms formation even after so many years of trauma.

## INTRODUCTION

Carotid injuries are difficult to evaluate and treat owing to very complex anatomy confined to a relatively narrow anatomic space and the hemodynamic status of the patient; other concurrent traumatic injuries also compromise the on-spot evaluation. Injuries to arteries of the thoracic outlet constitute 5–10% of arterial trauma.^1^ Pseudoaneurysm of the carotid artery is extremely rare and mostly are result of blunt or penetrating trauma.^2^ Although pseudoaneurysm formation has been typically reported secondary to blunt mechanisms; a delayed presentation (i.e. after 5 years) following a penetrating injury is much less described. We are reporting a unique case of saccular pseudoaneurysm of the mediastinal segment of the left common carotid artery in a patient with delayed presentation (10 years after trauma), and a penetrating and persistent foreign body in thorax as a predisposing factor. The delayed presentation is very rare, and only one such case has been reported,^3^ to the best of authors’ knowledge. Moreover, no case of carotid pseudoaneurysm is reported in the literature due to mechanical trauma by a retained foreign body. Only three cases of carotid aneurysm/perforation are reported due to ongoing trauma by hyoid bone^4^ which of course is not a foreign body.

## CASE REPORT

**Fig.1:**
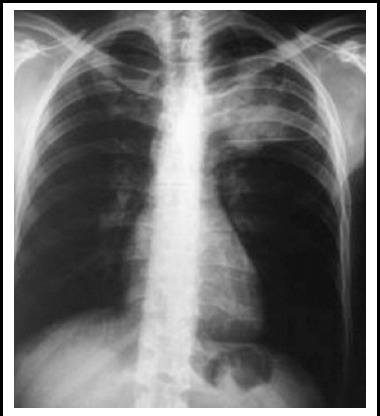
Chest x-ray shows soft tissue density in left upper lobe with a linear metallic foreign body along its inferior aspect.

**Fig.2:**
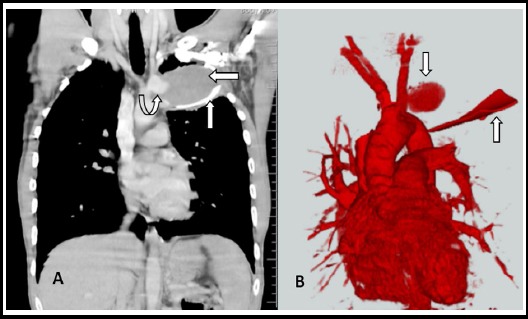
(A+B): MDCT of Chest (A) shows a saccular aneurysm of mediastinal part of left common carotid artery (curved up arrow) surrounded by thrombosis (left arrow) with a cylindrical linear metallic foreign body (up arrow) and CT Angiography. (B) confirmed a saccular aneurysm of left proximal common carotid artery (down arrow) and a cylindrical linear metallic foreign body (up arrow).

A 33-year man presented with hemoptysis through medical unit to our department in March 2017. He had history of penetrating trauma to the left upper chest by falling over a fluorescent tube light in 2006. He was treated at a local hospital, and was thought to have sustained superficial laceration without major vessels injury at that time. The skin was sutured and he remained asymptomatic afterwards. He had minimal hemoptysis on and off since 10-months whenever he had severe bout of cough due to any reason. There was no fever, chest pain, dyspnoea, hoarseness of voice, stridor or dysphagia. Physical examination was completely unremarkable. Patient was evaluated with chest X-ray for suspected tuberculosis, which showed an ill-defined soft tissue density in left upper lobe with a linear metallic foreign body (Fig.1). No evidence of adjacent rib erosion or expansion was there. Then contrast enhanced CT of chest performed which showed a saccular contrast filled out-pouching arising from mediastinal part of left common carotid artery measuring 2.3x3.5 cm about 1.2 cm above its origin. It was surrounded by a thrombus measuring 4.2x9.4 cm with a cylindrical linear metallic foreign body measuring 9.7cm in length and 3.3cm in caliber (Fig.2A). It was extending into the ipsilateral axilla through 2^nd^ intercostal space by eroding the anterior end of 2^nd^ rib. CT angiography (CTA) confirmed the diagnosis (Fig.2B). He was planned for endovascular repair with stenting which he could not afford due to financial constraints. He is currently on conservative follow up.

## DISCUSSION

The causes of carotid pseudoaneurysm are varied but trauma and prior carotid repair are the most common causes.^5^ Other causes include atherosclerosis, infection, and rare etiologies such as collagen vascular disease, fibromuscular dysplasia, and irradiation. Carotid injuries after blunt or penetrating trauma can result in so many complications like transection, pseudoaneurysm, arterio-venous fistula, dissection, occlusion or aneurysm formation. Development of pseudoaneurysm can take few hours to several years after initial arterial injury, normally presenting within five years,^3^,^5^ but our patient presented after 10 years of penetrating trauma over left chest. To best of authors’ knowledge, only one case of delayed presentation of pseudoaneurysm of cervical portion of left common carotid artery is reported in literature, presenting more than twenty years after self-inflicted neck injury in a known schizophrenic patient.^3^ Pseudoaneurysm of the common carotid artery related to ongoing trauma due to retained foreign body is extremely rare, and no such case is reported earlier. Only three cases of carotid aneurysm/perforation are reported due to trauma by hyoid bone.^4^

Extracranial carotid artery pseudoaneurysms and aneurysms are extremely rare, altogether accounting for only 0.4–4% of all peripheral artery aneurysms.^6^-^8^ Studies have shown the incidence of 0.02%-0.4% of carotid pseudoaneurysms among all trauma patients.^9^ Post-traumatic saccular carotid aneurysms most often involve the mid segment of the cervical internal carotid artery.^8^ Although fusiform enlargement of the mediastinal segment of the left common carotid artery can occur in atherosclerotic disease and in some types of aortitis but a saccular pseudoaneurysm in this location has been recorded only in a few instances after major blunt chest trauma.^8^

CT and MR imaging provide initial information about the pseudoaneurysm,^10^ but MR imaging of thorax is technically limited due to long imaging time and breathing artifacts, and usually performed with ECG-gated technique which is not easily available. Multidetector CT (MDCT) is more preferred due to easily availability, cost effectiveness and rapid scanning time. It is useful in making the diagnosis of aneurysm but also depicts the relationship of the aneurysm to the surrounding anatomical structures and displays intraluminal/extraluminal thrombotic material.^10^ So our patient underwent MDCT of chest that not only shows the aneurysm but also the surrounding thrombosis and retained foreign body as a predisposing factor. The minimally invasive imaging such as MR or CT angiography confirms the aneurysm, as seen in our patient’s CTA. Carotid angiography has diagnostic and therapeutic potential by endovascular intervention.^9^ This patient was advised Carotid angiography for endovascular stenting but he didn’t follow this advice due to lack of availability of such facilities at public tertiary hospitals and financial constraints precluding it from consulting private hospitals.

## CONCLUSION

The injuries of the carotid artery are uncommon but are serious consequences associated with either blunt or penetrating cervical trauma. The delayed presentation of carotid pseudoaneurysm after penetrating injury is extremely rare as exemplified by this case. Carotid artery stenting has emerged as a safe and effective alternative to surgical repair of carotid injuries but utility is limited by availability and cost of the procedure.

### Authors Contribution

**RS:** Concept, literature search, data collection, data interpretation, drafting, reviewing and finalizing the report.

**SS:** Concept, data interpretation, reviewing and final approval of the report.

**PAS:** Literature search and data collection.

**QN:** Literature search and data collection.
